# Radicalism

**Published:** 1920-03

**Authors:** F. C. Brush

**Affiliations:** New York City


### RADICALISM

BY F. C. BRUSH, D.D.S., NEW YORK CITY

A retrospective view of the contents of dental journals of the past few years leads to the conclusion that dentistry has not been exempt from the infection of radicalism that seems to have tainted our whole social structure. Instead of finding carefully considered and conservative statements as expressed by the leaders of the past, we find the most radical views voiced with positive assurance by men whose chief claim to fame lies in the ability to talk upon any subject at any and all times.

Not infrequently we come across the statement made or implied that unless some particular method of procedure is adopted forthwith one will be liable to the charge of malpractice. In fact, malpractice seems to have become a word of quite common usage, not only at dental meetings, but in conversations with patients and the general public. This has had an effect that might have been expected, even if not desired, for insurance men say that suits for malpractice are increasing rapidly, and the time has almost come when a man can not be said to have arrived in the profession unless he has had one or more such suits to defend.

The insurance men also say that upon investigation it is generally found that the reason for the bringing of the suit can be traced to the psychological effect of some unwise statement or accusation made by some follower of these radical talkers.

THE DANGER OF DEVITALIZED TEETH EXAGGERATED

The prevailing idea that non-vital teeth are the initial cause of about all of the "ills that flesh is heir to" must certainly be classed as a radical one. The wholesale devastation that is following in its wake is appalling to men of calmer judgment. Now that a little time has elapsed, giving a better perspective, and results can be checked up, it appears that devitalized teeth which have been treated, the canals filled to and through the apex, by methods that are being so insistently heralded these days, do not show a far different percentage of successes and failures than those which were handled with an equal amount of care and filled by some of the older methods.

Probably every dentist who has been long in practice and has kept detailed records can recall numerous cases where teeth that have been devitalized and treated years ago, according to methods then in vogue, have gone for years without showing the slightest symptom of disease, having to all intents and purposes fulfilled their functions in a sufficiently healthy manner, and many of them still remain in this condition.

Such records will also reveal instances where teeth that have been treated in a like manner have remained for years in a satisfactory condition, without showing any symptoms that would arouse suspicion that all was not well, and then suddenly, without apparent cause, have developed an acute alveolar abscess. I say without apparent cause for this reason: Dentists as a rule confine themselves strictly to their own field and seldom investigate the general condition of the patient, and at such a time, for the purpose of seeking a possible exciting cause in some other portion of the body, the need of a thorough physical examination has not been thought of or at least practiced.

The point of the matter is, if it is possible for bacteria to be carried in the blood stream from the apex of a tooth to some other part of the body and there set up a local disturbance, as is now being claimed, is it not equally possible for the same blood stream to convey bacteria from some remotely situated diseased tissue to the apex of a tooth whose resistance has been lowered by the loss of its vitality, and there cause an acute abscess to develop? In other words, may it not be possible that an abscess at the apex of the root of such a tooth might be an effect due to a diseased condition elsewhere, rather than being the exciting cause of the physical condition? How else can we account for phenomena that are familiar to all of us?

The usual experience has been that an infection makes its presence known almost as soon as it occurs. Is it reasonable, therefore, to suppose that a tooth which is infected will remain apparently healthy and quiescent for years before suddenly developing an abscess? When an abscess does occur, after a long period of evident health, is it not reasonable to suspect that the infection may have been contributed from some other diseased tissue? Consider the Roosevelt case, for instance. When it was boldly announced that his illness and death were due to an abscessed tooth of twenty years' standing, the public mind was ready to accept such a tale, and it made a good theme for a *Ladies' Home Journal* story.

If some of the facts in the case are investigated, however, it will be found that Mr. Roosevelt had indulged to excess in violent physical exercise nearly all his life; that in the Santiago campaign he contracted Cuban fever, and that for years he suffered from recurrent attacks (spoken of frequently in letters to his children); that long after he had passed middle life he took up Japanese wrestling, and his muscles were severely strained and his body badly bruised; that on his African hunting trip he was bitten by tropical insects, and his diary at the time records that day after day he suffered from fever; that on his journey into the Brazilian wilderness he was again bitten by poisonous insects and contracted jungle fever (from which a white man seldom recovers), at the same time undergoing deprivation and violent physical exertion. He returned home broken in health, and was a constant sufferer in one way or another from that time on. Boils and abscesses occurred at various times; sciatic rheumatism developed, and several operations were undergone. Some months before his death he was told that he would probably have to spend his days confined to a chair. And when the call came, in his sleep, it was an embolism that was the immediate cause of his death. Yet in spite of all this there are some so bold as to declare that his illness and death were due to an abscessed tooth.

### THE UNCERTAINTY OF THE RADIOGRAPH

Concerning the X-ray and the wonderful tales it is supposed to tell about hidden conditions. It is quite possible to play tricks on the X-ray and the expert who claims to interpret the radiographs. To do so is both amusing and instructive, but it tends to shake one's faith somewhat in both of them. How is it that a dark area or shadow about the apex of a tooth is almost invariably interpreted as an abscess, or an infected area, even when there are no other symptoms to corroborate it, when teeth that are known to be unquestionably abscessed will sometimes show a clear normal condition in the radiograph? That devitalized teeth with empty canals will be interpreted as being vital teeth? That canals which are filled with various kinds of dressings will be reported as being left unfilled? When a radiograph shows a dark area supposedly indicative of tissue destruction, can the expert honestly say whether it is or was an abscess, basing his judgment on the mere evidence of the radiograph alone?

A case taken at random from my records: A crowned upper canine was radiographed by an expert, who diagnosed the trouble as an abscess and recommended apicoectomy. A careful examination with a probe revealed that the root was split its entire length, and when the tooth was extracted there was no sign of an abscess.

Another case: The expert radiographer at a prominent medical institution made a diagnosis of a large abscess involving the area around two teeth, and was rather insistent that an operation should be performed almost immediately. However, it was merely necessary to remove the two artificial teeth, and the suspected abscess disappeared. Had the patient mentioned to the expert the presence of the artificial teeth a ridiculous mistake might have been avoided. Such mistakes would be good jokes if it were not for the serious psychological effects upon susceptible people.

There are any number of cases where apparently healthy teeth have been radiographed and the negative has shown shadows or dark areas about the roots, yet when these same teeth, twenty-four or forty-eight hours later, have again been radiographed from a slightly different angle, the negative has not shown even a trace of a shadow. To have operated on such a tooth on the mere evidence of the first radiograph would be radicalism; better judgment would deem it unwise to condemn such a tooth until more definite evidence could be obtained.

The foregoing remarks are not made with any intention of disparaging the real value of the X-ray as an aid in diagnosis, but rather to show that it is unwise to take too much for granted or to make snap judgments; in other words, not to be too radical. The wonder lies not in how much some of us know, but in how much we know that is not so.

Another common source of trouble is when some amateur radiographer takes a radiograph of a root with a doweled crown. The dowel, extending part way in the canal, will stand out boldly and clearly in the negative, which is shown the patient. While explaining the details a more or less casual remark is made that the canal has not been filled to the apex, that such a condition may prove detrimental to the health, together with a veiled allusion to malpractice. If the patient happens to be one of those whose chief indoor sport is starting suits for damages, some one is more than likely to have such a suit to defend. Having been led to believe that the X-ray tells the whole truth and nothing but the truth, it would be hard to convince the patient or a jury that the tooth had been properly cared for in a conservative way, and in a manner that was seemingly for the best interests of the patient, all things being considered.

Disputes of this kind not only engender hard feelings between members of the profession, but also tend to cause the public to lose confidence in and to question the honesty and ability of medical men in general. It is altogether too common a practice nowadays for physicians to take radiographs of the teeth, pass judgment, and order extractions, without consulting with a dentist about the case. This procedure is just about as radical as it would be for a dentist to order an operation on some other part of the body in the hope of relieving a condition manifested in the mouth.

Recently there have come to my attention the cases of five women who have undergone the operation for complete hysterectomy. Each one is complaining of painful knee-joints accompanied with stiffness and considerable enlargement. One of them followed the advice of a physician and had a number of teeth extracted, without any apparent improvement in her condition. Another, before following similar advice, consulted a dentist; the teeth and other tissues of the mouth were found in a normal condition. Another has a normal mouth with no non-vital teeth. I can not say as to the condition of the mouths of the remaining two.

What is to be inferred from cases like these? Is it merely a coincidence that all of them have the same symptoms under the same bodily conditions? May it not be possible that the complete removal of one function has necessitated a readjustment which has manifested itself by this local disturbance in the knee-joints? If so, would it not be a little less radical to consider and investigate this possibility before ruthlessly sacrificing inoffensive teeth?

All this goes to show that what is most to be desired is less radicalism and a good deal more plain reasoning and common sense on the part of dentists, with closer co-operation between them and physicians if the best interests of patients are to be served and confidence in the professions is to be restored to anything like its old position.

—*The Dental Cosmos.*

